# The nutritional and hedonic value of food modulate sexual receptivity in *Drosophila melanogaster* females

**DOI:** 10.1038/srep19441

**Published:** 2016-01-18

**Authors:** Jenke A. Gorter, Samyukta Jagadeesh, Christoph Gahr, Jelle J. Boonekamp, Joel D. Levine, Jean-Christophe Billeter

**Affiliations:** 1 Groningen Institute for Evolutionary Life Sciences, PO Box 11103, University of Groningen, Groningen, 9700 CC, The Netherlands; 2 Department of Biology, University of Toronto at Mississauga, 3359 Mississauga Road, Mississauga, ON, L5L 1C6, Canada

## Abstract

Food and sex often go hand in hand because of the nutritional cost of reproduction. For *Drosophila melanogaster* females, this relationship is especially intimate because their offspring develop on food. Since yeast and sugars are important nutritional pillars for *Drosophila,* availability of these foods should inform female reproductive behaviours. Yet mechanisms coupling food and sex are poorly understood. Here we show that yeast increases female sexual receptivity through interaction between its protein content and its odorous fermentation product acetic acid, sensed by the Ionotropic odorant receptor neuron Ir75a. A similar interaction between nutritional and hedonic value applies to sugars where taste and caloric value only increase sexual receptivity when combined. Integration of nutritional and sensory values would ensure that there are sufficient internal nutrients for egg production as well as sufficient environmental nutrients for offspring survival. These findings provide mechanisms through which females may maximize reproductive output in changing environments.

The act of mating may be costly to *Drosophila* females as it has been shown to reduce their lifespan, increase the risk of infection and cause physical damage^1^,^2^,^3^,^4^. Because of these costs, females are predicted to determine an optimal number of copulations based on maximizing the number of offspring they produce in a given environment^5^,^6^,^7^,^8^,^9^,^10^. Food availability is paramount because it provides the energy for both the production of eggs and the survival of offspring after birth. Therefore, production of offspring puts high nutritional demands on females, which explains the close association often observed between sexual activity and food availability^11^. In mammals, poor nutritional conditions delay the onset of sexual activity in malnourished prepubescent females, reduce ovulation rate and can even result in termination of pregnancy in case of acute food shortage^12^,^13^. This shows the existence of mechanisms preventing reproduction in conditions unsupportive of offspring survival. Food availability fluctuates periodically during the year and many animals use photoperiod to be reproductively active during seasons when food is plentiful^14^. However environmental conditions can be unpredictable, because they vary stochastically, necessitating animals to develop mechanisms for adapting their reproductive output to acute fluctuations in food availability. The ability to sense when nutrition is available and couple it with sexual activity would provide a mechanism to maximize reproductive output in varying environments. Yet mechanisms allowing such coupling of food availability and reproduction are poorly understood.’

The fruit fly *Drosophila melanogaster* has long been used to understand the mechanisms controlling reproductive behaviours^15^. *D. melanogaster* has a strong bond with yeast, which is an important protein source in its natural habitat and is necessary for the full development of its offspring^16^. Volatile yeast fermentation products such as ethyl acetate^16^,^17^,^18^ are attractive to flies and promote aggregation. Yeast-derived products activate several classical Odorant receptors (Or) in the fly olfactory system, which mediate attraction to yeast^18^,^19^,^20^. Once on their substrate, the courtship intensity of male fruit flies towards females is enhanced by the presence of food through sensing of the fruit odour phenylacetic acid^21^. This compound is sensed via the Ir84a receptor, which belongs to a second family of odorant receptors called Ionotropic receptors (Ir)^22^. Although these mechanisms exemplify the intimate relationship of food and sex for males, it seems likely that female reproductive behaviour should be under nutritional influence as well. Females bare most of the energetic cost of reproduction and use yeast as a source of protein for ovary maturation and egg production^5^,^23^,^24^,^25^. Indeed females mate more frequently and are more fecund under conditions of high yeast abundance than when yeast is lacking^6^,^26^,^27^,^28^, suggesting that nutritional yeast availability directly or indirectly stimulate female sexual receptivity and fecunidty. Despite the importance of yeast for female reproduction, no direct effect of yeast on female sexual receptivity has been documented and no mechanisms for how nutritional conditions may be evaluated have been proposed. In this study, we investigated the influence of the nutritional environment, and yeast in particular, on *D. melanogaster* reproduction; focusing on female sexual receptivity and progeny production to understand the mechanisms coupling evaluation of food availability with reproduction.

## Results

### Substrates containing yeast or sugars increase receptivity and production of offspring

**Figure 1:**
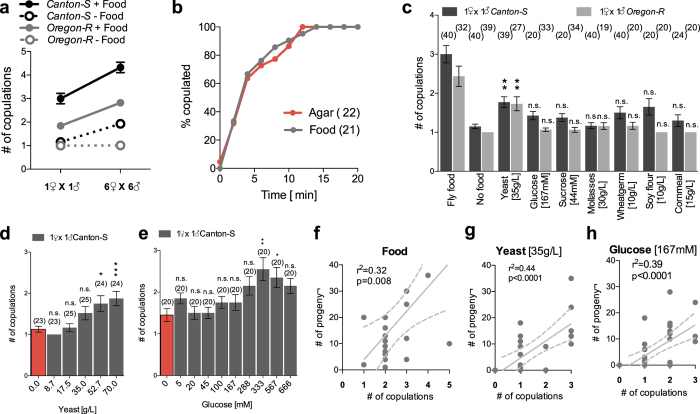
Food conditions influence receptivity and offspring production. (**a**) Mean number of copulations over a 24-hr period per female in pairs consisting of 1 female and 1 male or groups of 6 females and 6 males of the indicated strain. Flies were assayed either in the presence of fly food “Food” or with 1% agar in water “No Food”. Number of replicates ranges from 19–46. Error bars indicate Standard Error of the Mean (SEM). (**b**) Cumulative percentage of pairs of virgin male and female *Canton-S* that copulated on substrates either containing food or no food over 20 minutes. Mating latency in the two conditions did not significantly differ (t-test with Welch’s correction: p = 0.199). Number of replicates is indicated between parentheses. (**c**) Effect of fly food ingredients on copulation. Number of copulations of full fly food (red bar) is indicated for comparison but not included in the analysis. Statistical analysis reveals a significant effect of food ingredients on copulations in both *Canton-S* (Kruskal-Wallis test; p < 0.001) and *Oregon-R* (Kruskal-Wallis test; p < 0.001). Differences between “No food” treatment and individual food ingredients within strain were tested using Dunn’s post-hoc test, whose resulting p values are reported above the graph: *p < 0.05; **p < 0.001; ***p < 0.0001. n.s. = non significant. (**d**) Dose response curve of yeast effect on copulation in *Canton-S*. Statistical analysis reveals a significant effect of yeast dose on copulations (Kruskal-Wallis; p < 0.0001). Differences between no yeast (0 g/L) treatment and individual yeast doses were tested using Dunn’s post-hoc test, whose resulting p values are reported above the graph as in (**c**). (**e**) Dose response curve of glucose effect on copulation in *Canton-S*. Statistics are as in (**c**). (Kruskal-Wallis; p = 0.0006). (**f–h**) Correlation between the number of copulations and the number of progeny produced on different food substrates. Dots represent individual females. Slopes represent linear regressions and are significantly non-zero on all three substrates (p-value on graphs). Correlation between the number of copulations and progeny is significant for all three substrates: Spearman correlation; Food, p = 0.038; Yeast, p = 0.002; Glucose, p < 0.0001. Dashed lines represent 95% confidence intervals. See Table S1 for full statistics.

*D. melanogaster* aggregate on food substrates through their common attraction to yeast volatile fermentation products^15^. The increase in group size driven by food attraction could directly promote sexual activity. To test the effect of group size on reproduction, we housed males and females in either pairs or in groups of six males and six females and observed their reproductive behaviour on a fly food substrate containing yeast for 24 hours. Pairs and groups were assayed at similar density by using different size mating arenas. Individual flies housed in groups copulated significantly more than those housed in pairs showing that group size can modulate female receptivity (Fig. 1a, main effect of group size: p < 0.0001; Table S1). The effect of yeast as an aggregation factor^16^ may thus indirectly increase reproduction by increasing group size. However, the number of copulations fell down dramatically in both pairs and groups when food was removed from the substrate (Fig. 1a, main effect of food: p < 0.0001; Table S1), indicating that sexual receptivity is also directly influenced by food. These effects are significant for the two wild type strains tested (*Canton-S* and *Oregon-R*), although these two strains differ in their general level of female receptivity (Fig. 1a, main effect of genotype: p < 0.0001; Table S1). Absence of interaction between genotype and group size in the presence (p = 0.796) or absence of food (p = 0.995) indicates that the effect of food or group size in not affected by the strain of flies studied (Fig. 1a; Table S1).

As food directly increases the number of copulations, we investigated at what stage of reproduction this effect takes place. We first tested the effect of food availability on wild-type virgin sexual behaviour by measuring the latency to mate on an agar substrate with or without Fly food. The presence of food and the associated yeast did not significantly affect mating latency, indicating that food does not overtly influence virginal sexual activity (Fig. 1b).

It has previously been shown that females favor eating yeast only once they have mated^29^. The influence of food containing yeast may thus reveal itself after the first mating. We monitored the number of copulations in pairs of males and females housed on different food substrates in two different wild-type strains. Flies housed with food mated from two to three times in 24 hours, while flies housed in absence of food mated only once (Fig. 1c). Individual ingredients of fly food induced a lower number of copulations than on full fly food, and only yeast significantly elevated number of copulations over the “no food” condition (Fig. 1c). It did so in flies from both the *Canton-S* and *Oregon-R* strains indicating the general relevance of yeast for mating in *Drosophila melanogaster*.

Other ingredients such as glucose modestly increased copulation, without reaching significant effects (Fig. 1c). To test the importance of the dosage of ingredients, we established a dose response of females’ response to yeast (Fig. 1d) and glucose (Fig. 1e) to determine at which concentration these foods effectively affect mating. The number of copulations increased as yeast concentration increased (Fig. 1d) suggesting that the amount of copulations is tuned to yeast concentration. For glucose, the number of mating increased with concentration up to 333 mM, but decreased thereafter indicative of a negative consequence of high glucose concentration perhaps connected to high osmosis. Dose-dependent effect on copulation suggests that flies gauge food resources and modulate the number of copulations accordingly (Fig. 1d,e).

Finally, we tested the hypothesis that females that copulate more also have higher fecundity by assessing the correlation between number of copulations of a female and the amount of eggs laid during the 24 hour of the assay that produce adult progeny. We found that females who mate more also have more offspring (Fig. 1f). This correlation remains significant for substrates containing fly food, yeast or glucose (Fig. 1f–h). We conclude that *D. melanogaster* modulates sexual receptivity in response to food availability and that this may impact their fecundity.

### The smell of yeast increases copulation via ionotropic receptors of the female olfactory system

Our data indicate that yeast is a major food factor influencing *D. melanogaster* reproduction. Yeast could influence reproduction through a variety of mechanisms. The smell of yeast may signal a favourable environment for offspring development directly increasing females’ willingness to mate. Alternatively, females consuming yeast acquire amino acids for egg production, which may indirectly feed back on mating receptivity. Finally, yeast may act specifically on males to increase courtship, as previously demonstrated^21^, resulting in increased female sexual receptivity.

**Figure 2:**
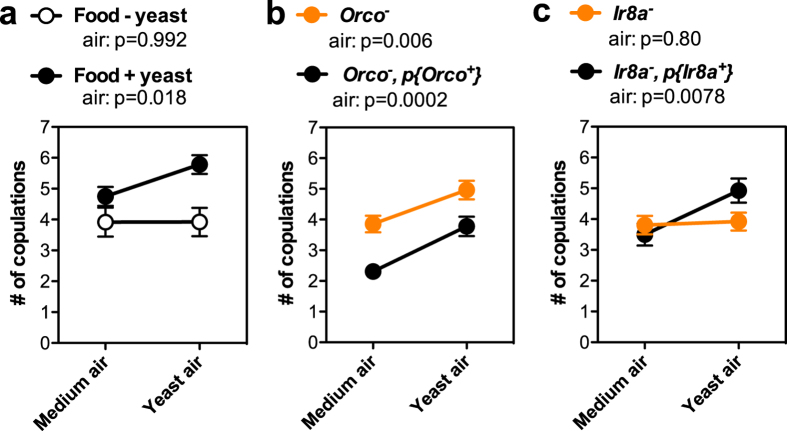
Smell of yeast affects female sexual receptivity via the ionotropic odourant receptor family. (**a**) Mean number of copulations of one female and one male *Canton-S* exposed to yeast vapour and/or nutritional yeast over 24 hr. Air was bubbled either through sterile yeast medium “Medium air” or through a yeast culture “Yeast air”. Yeast was subtracted from, “Food − yeast” (White dots), or added, “Food + yeast” (Black dots), to the fly food recipe. Number of pairs tested ranged from 12–24. (**b**) Mean number of copulations of one *Orco*^−^ or one *Orco*^−^ with a genomic rescue construct (*Orco*^−^*, p{Orco*^*+*^**}) female housed with one wild-type *Canton-S* male. Yeast was included in the food. Yeast and medium air conditions were tested as in (**a**). The number of pairs tested ranged from 27–29. (**c**) Mean number of copulations of one *Ir8a*^−^ or one *Ir8a*^−^ with a genomic rescue construct (*Ir8a*^−^*, p{Ir8a*^*+*^**}) female housed with one wild-type male tested as in (**b**). Number of pairs tested ranged from 25–27. Error bars indicate S.E.M. The post-hoc effect of air within one food condition or genotype was tested using mixed effect models, p values are reported above the graphs. See Table S1 for full statistics of main effects and post-hoc models.

We tested the hypothesis that the smell of yeast directly influences female sexual receptivity. To supply yeast odour, air was bubbled through a live yeast culture, or through sterile yeast medium in case of control air, and vented into mating assay dishes containing fly food with or without yeast added. A wild-type male and female were exposed to these conditions for 24 hr and the number of copulations was monitored. In this experiment the most important factor is the presence of yeast in the food, since the number of copulations is increased when the food contains yeast whether or not yeast air is also provided (Fig. 2a; main effect of food: p = 0.001; Table S1). Air, bordering significance, has an effect on number of copulations as well (air: p = 0.061; Table S1). Post-hoc analysis on this effect shows that females tested on food with yeast increase copulation in response to the odour of yeast, but females tested on food without yeast do not convincingly show such response (Fig. 2a; See Table S1 for details on effect estimates). We conclude that yeast airborne compounds increase sexual receptivity, but only when yeast is also available in the substrate.

To determine whether female sexual receptivity is directly influenced by the smell of yeast, we blocked the female’s, but not the male’s, ability to smell. Attraction to yeast is mediated by classical odorant receptors (Or)^18^,^19^,^20^, which require the Orco co-receptor function^30^. To test the importance of yeast smell specifically on female sexual receptivity, *Orco*^−^ mutant females paired with wild-type males were exposed to yeast air or control media air in the presence of food containing yeast. Yeast air significantly increased number of copulations in both *Orco*^−^ and *Orco*^−^ rescue females compared to media air (Fig. 2b, air: p = 0.001), indicating that Ors are not necessary to increase female sexual receptivity in response to yeast air (Fig. 2b). *Orco* however is not required for the function of Ionotropic Receptors (Ir), a second family of odorant receptors expressed in different olfactory neurons^22^. We tested females mutant for *Ir8a*, a gene encoding a co-receptor necessary for the function of half of the Irs, including those mediating the olfactory response to several identified yeast fermentation products^31^. Comparing *Ir8a*^−^ mutant females and their rescue counterparts in relation to absence or presence of yeast air shows an interaction between air and genotype indicative of the necessity of *Ir8a* for females to respond to the smell of yeast (Fig. 2c, interaction air by genotype: p = 0.047). Indeed, *Ir8a*^−^ mutant females did not copulate more when exposed to yeast air compared to media air showing that *Ir8a* is necessary for yeast sensing (Fig. 2c). Response to yeast air was restored in females in which *Ir8a*^−^ was rescued by an *Ir8a* genomic construct (Fig. 2c). These data clearly demonstrate that the Ir8a channel of the female olfactory system mediates sensing of aphrodisiac yeast airborne compounds that stimulate female sexual receptivity. Because we only manipulated female genotype in these experiments, these data also imply that female increase sexual receptivity in response to yeast independently of increased male courtship in response to food odours^21^.

### Females modulate receptivity and fecundity through an interaction between acetic acid and amino acids sensing

**Figure 3:**
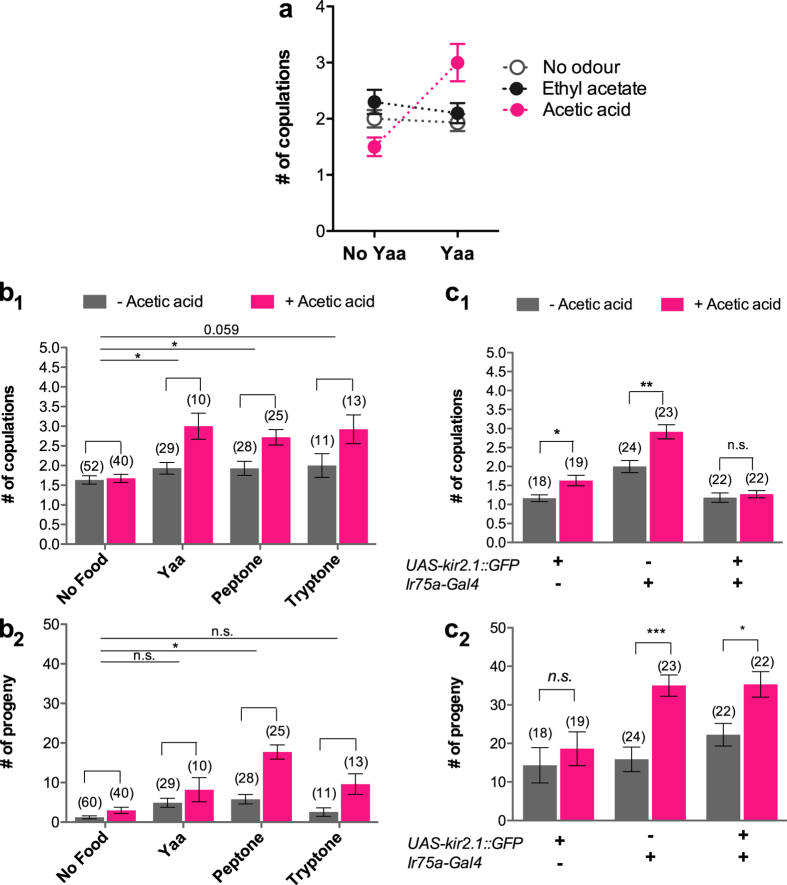
Acetic acid and proteins produced by yeast interact to modulate female reproductive behaviours. (**a**) Mean number of copulations of one *Canton-S* male and female exposed to the indicated odorants (concentration of 1% v/v) in presence or absence of Yeast amino acids (Yaa). Number of replicates ranges from 10–29. Error bars indicate S.E.M. (**b**) Mean number of copulations (**b**_**1**_) and progeny (**b**_**2**_) of one wild-type male and female exposed to a protein source with or without Acetic acid (1% v/v). Yeast amino acids “Yaa”, Peptone and Tryptone are at a concentration of 30 g/L. The effect of Acetic acid within one amino acid source condition was tested using GLM, whose resulting p values are reported above the bar graphs: n.s. (non significant); *p < 0.05; **p < 0.001; ***p < 0.0001. Number of pairs tested for each condition is indicated above each bar graph. (**c**) Mean number of copulations (**c**_**1**_) and progeny (**c**_**2**_) of one wild-type male and one female of the indicated genotype exposed to Peptone [30 g/L] with or without the addition of Acetic acid (1%v/v). The effect of Acetic acid within one genotype was tested using mixed effect models, resulting p values are reported above the graphs. See Table S1 for full statistics.

The smell of yeast increases number of copulations only when yeast is present in the food substrate. This suggests that hedonic (sensing of the yeast non-nutritious odorants) and nutritional contents work in combination to affect female receptivity. To test this hypothesis we investigated whether the amino acid content of yeast (50% of its dry weight) alone or when coupled with specific yeast-derived odours can increase copulations. Flies were housed on a substrate either supplemented with a mixture of free amino acids at the ratio found in yeast (Yaa) or not (no Yaa)^24^. In addition, two major yeast odorant products, ethyl acetate and acetic acid^16^, were individually added to the substrate. Overall there was no significaint effect of food substrate (main effect food: p = 0.1847; Table S1) or odorant product (main effect air: p = 0.302; Table S1), neither Yaa nor any of the odorant products were individually able to increase number of copulations. The number of copulations was, however, significantly affected by an interaction of food substrate and odorant product (interaction food by odorant product: p < 0.001; Table S1). On a quantitative level, it is clear that only acetic acid (as compared to ethyl acetate or control air, Table S1) that only acetic acid significantly increases number of copulations when Yaa is present in the substrate (Fig. 3a) (food by odorant product interaction: acetic acid *vs* ethyl acetate: p = 0.001 and acetic acid *vs* control; p < 0.001; Table S1). This shows that flies need to simultaneously sense amino acids and acetic acid, but not ethyl acetate, to be sexually aroused (Fig. 3a). We conclude that flies integrate the amino acid content of yeast with specific yeast odorants to modulate sexual receptivity.

To test the general ability of amino acids to influence female sexual receptivity and fecundity in combination with acetic acid, we also tested peptone and tryptone, which are mixes of peptides and amino acids derived from enzymatic digestion of animal proteins and casein, respectively. None of the amino acid sources alone resulted in more copulations (Fig. 3b1) or progeny produced (Fig. 3b2) compared to “No food” control (see Table S1 for statistics), confirming that amino acids are not sufficient to modulate female reproductive behaviours. All three amino acid sources increased copulation when combined with acetic acid (Fig. 3b1)(interaction food by acetic acid: p = 0.003; Table S1). Also, in overall, production of progeny was increased when combined with a food source of amino acids (Fig. 3b2, Interaction food by acetic acid: p < 0.001; Table S1), but this effect can only be significantly ascribed to peptone (Fig. 3b2; interaction food peptone by acetic acid *vs* no food by acetic acid: p = 0.019; Table S1). These data together show that flies need to simultaneously sense amino acids and acetic acid to modulate reproductive behaviours. This supports our hypothesis that the hedonic and nutritional value of yeast interact to modulate reproductive behaviours.

To test whether the effect of acetic acid on copulation is mediated by female olfaction, we assayed females in which the Ir75a olfactory sensory neuron is silenced by the expression of the Kir2.1 inward rectifier potassium channel^32^. Calcium imaging studies have previously implicated Ir75a with the sensing of acetic acid^33^. Moreover, Ir75a function is blocked in *Ir8a*^−^ mutants^31^, which we showed to be necessary for the effect of yeast air on mating (Fig. 2c), making Ir75a-expressing neurons a candidate channel for the effect of yeast-derived odorants. Experimental females were housed with wild-type males in mating arenas layered with agar containing peptone, with or without acetic acid. Silencing Ir75a neuron inhibited increased copulations in response to acetic acid (Fig. 3c_1_) indicating that the smell of acetic acid is stimulatory to mating. Fecundity was not affected by blocking Ir75a neuron (Fig. 3c_2_) in keeping with a previous report showing that acetic acid directly promotes egg laying via gustatory and not olfactory receptors^34^. This result indicates that increased sexual receptivity and increased fecundity, although correlated, can be mechanistically uncoupled where fecundity can get increased in the absence of increased mating.

### The hedonic and nutritional value of sugar interact to determine sexual receptivity

**Figure 4:**
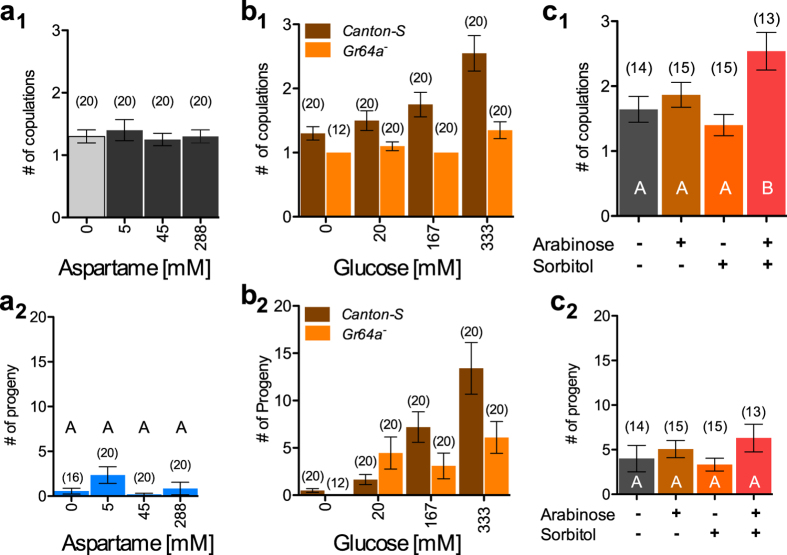
Taste and calorific value of sugars interact to modulate female reproductive behaviours. (**a**) Mean number of copulations (**a**_**1**_) and progeny (**a**_**2**_) of one wild-type female housed with a wild-type male. The sweeteners Aspartame was added to the substrate at the indicated concentrations. Error bars indicate S.E.M. (**b**) Mean number of copulations (**b**_**1**_) and progeny (**b**_**2**_) of a *Gr64a*^−^ mutant female or a wild-type *Canton-S* female housed with a wild-type male. The Petri dish was layered with increasing doses of glucose. (**c**) Mean number of copulations (**c**_**1**_) and progeny (**c**_**2**_) of one wild-type female housed with one wild-type male on a substrate containing the carbohydrate arabinose (200 mM) and the taste-less carbohydrate sorbitol (200 mM) singly or in combination. The control treatment was housed on an agar only substrate. Effects of different carbohydrates were compared to the agar control using mixed effect models. Bar graphs labeled with same letters are not significantly different from each other. The number of pairs tested is indicated above the bar graphs. See Table S1 for full statistics.

Yeast modulates female receptivity through an interaction between its hedonic and nutritional value. To test whether this interaction is a common theme in the modulation of reproduction by food, we investigated whether the effect of glucose on copulation (Fig. 1e) can too be broken down into a hedonic and a nutritional component. Sweeteners have little nutritional value but are ingested and perceived as sweet by flies at concentration as small as 2 mM^35^. We therefore tested the sweetener aspartame to determine whether the hedonic value of sugar is sufficient to modulate the number of copulations. Aspartame had no effect on number of copulations or production of offspring (Fig. 4a_1–2_, main effect aspartame on number of copulations; p = 0.973 and offspring production; p = 0281, see Table S1M). Two additional sweeteners, sucralose and saccharine, also had no effect (Supplementary Fig. S1). The hedonic value or “sweetness” of sugar is thus not sufficient to affect number of copulations and production of progeny. We next tested the effect of the nutritional value of glucose on female receptivity and production of progeny. For this we assessed females mutant for the *Gr64a* taste receptor, which is essential for glucose tasting^36^, housed with wild-type males. Glucose increased the number of copulations (Fig. 4b_1_; glucose concentration: p < 0.0001) and production of progeny (Fig. 4b_2_, glucose concentration: p < 0.0001, Table S1) in wild-type females. However *Gr64a*^−^ mutant females only modestly responded to glucose by increasing copulation (glucose concentration: p = 0.023) and progeny production (glucose concentration p = 0.021) at the highest concentrations and did so only modestly (Fig. 4b_1–2_; Table S1). In *Gr64a*^−^ females glucose has a weaker effect compared to wild-type females demonstrated by a nearly two fold higher effect size of glucose on mating in *Canton-S* (Cohen’s r = 0.41) than in *Gr64a*^−^ females (Cohen’s r = 0.25). These data indicate that tasting glucose or getting its nutritional value is necessary but not sufficient for affecting female sexual receptivity and fecundity (Fig. 4b_1–2_). The hedonic and nutritional values of glucose thus interact to modulate female reproduction.

We confirmed the interaction of the hedonic and caloric value of sugar in a second experiment. The sweet tasting carbohydrate arabinose, which has no caloric value to flies, was provided either alone or in combination with sorbitol, which has caloric value but no taste^37^,^38^,^39^. Neither arabinose nor sorbitol affected copulation compared to agar control but they increased the number of copulations when added in combination (Fig. 4c_1_). This combination however did not have a significant effect on progeny production (Fig. 4c_2_). This again suggests that sexual receptivity and fecundity are not mechanistically coupled. Our data, however, clearly indicate that sugar increases sexual receptivity through interaction of its hedonic and caloric value analogous to the effect of yeast.

## Discussion

Here we investigated the influence of nutritional environment on *D. melanogaster* female reproduction. This study yielded three main insights. Firstly, we demonstrated that rich nutritional environments increase female sexual receptivity as well as the production of progeny. Females especially regard the presence of yeast as a rich nutritional environment. Secondly, to respond to this nutritional environment, females do not rely solely on one sensory modality, but they require perception of both the hedonic value (smell or taste) of food as well as its nutritional content. The specific yeast odour acetic acid, which is sensed by the ionotropic receptor Ir75a, is only sufficient to influence female reproduction when yeast amino acids are present in the food substrate. Sugar, on the other hand, is recognized by its sweet taste, which is sensed by the gustatory receptor Gr64a, and caloric content. And lastly, we showed that even though changes in female sexual receptivity and production of progeny are associated, they do not mechanistically depend on each other.

Our behavioural data show that modulation of female reproduction depends on the integration of two sensory modalities, perception of hedonic value and nutritional content. For the former we have provided insight into the mechanism by which both yeast and sugar are perceived. However, for the second part, we have not been able to propose any mechanisms. There are two possible hypotheses on what mechanisms might underlie the perception of nutritional content. The first is a second path of sensory perception; taste neurons tuned to nutrients would sense the nutritional value of food at the moment of ingestion and this would be integrated with the perception of smell or taste. Unfortunately, this hypothesis cannot be currently tested as gustatory receptors for amino acids have not yet been identified despite strong evidence of their existence^40^. If this hypothesis is correct, it depicts a risky strategy that does not insure against the inability of the peripheral nervous system to discriminate substances that smell or taste like food but that cannot be metabolized to fuel egg production. We, therefore, favour a second hypothesis where females assess the hedonic and nutritional value of food by integrating external (pre-ingestive sensory) and internal (post-ingestive sensory) processes. Such integration would allow gauging the available internal nutritional reserves of a female against environmental resources detected by the peripheral nervous system, thus signaling the normal requirement for mating in response to dietary factors that limit female reproduction. The existence of an internal amino acid sensor has been revealed by a change in feeding preference toward amino acids when deprived of this nutrient^29^,^40^,^41^. However, the cellular identity of amino acid sensors and the detailed molecular mechanisms are still unknown. The effect of glucose on female receptivity could putatively be mediated by very similar mechanisms, coupling peripheral external sensing of glucose with internal sugar sensing. Firstly, the fructose receptor Gr43a, expressed in six central brain neurons, serves as an internal indicator for the consumption of nutritious sugars^42^. Glucose is converted into fructose in the hemolymph after ingestion, and this fructose is then used as an internal readout of glucose ingestion^42^. Secondly, the insulin pathway is involved in female receptivity^10^ and could signal glucose reserves in the fly. How these findings are related to female reproduction remains to be investigated. It is clear, however, that female reproduction provides a system for the understanding of mechanisms that integrate external and internal nutrient sensing in a context that is relevant to the animal’s ecology.

Although female receptivity and fecundity are expected to be evolutionarily tightly linked, it is not surprising that the mechanisms regulating these behaviours can be uncoupled. Female receptivity is sometimes negatively correlated with production of offspring, as is the case in the post-mating response of *Drosophila melanogaster* females, whereby mating increases ovulation but reduces receptivity^43^. This negative correlation can be uncoupled through genetic manipulation^44^ or age^45^. These data together with ours show that female receptivity and production of offspring are regulated through partially distinct pathways, though in response to natural environmental stimuli they generally act in a coupled fashion. Both increases in female receptivity and production of offspring in response to rich nutritional environments lead to increased female fecundity. The ability to regulate these behaviours through different pathways might enable females to increase fecundity even in the absence of further mating opportunities^46^. Mechanistic uncoupling of these two reproductive behaviours may therefore serve an evolutionary purpose to ensure increased female fecundity in nutritionally rich environments.

In summary, here we have shown that the nutritional environment of *D. melanogaster* females modulates their reproduction and that females do not rely on one sensory modality for this, but require sensing the hedonic value as well as the nutritional content of food. Such integration could make sure that there are sufficient internal nutrients for egg production as well as sufficient environmental nutrients for offspring survival. Integrating information about environmental and acquired food resources may ultimately maximize reproductive output in a given environment.

## Methods

### *Drosophila* stocks and Genetics

The wild-type strain used for experiments were *Canton-S and Oregon-R. Gr64a*^2^ ^30^,^36^ mutant flies were placed into the *Canton-S* genetic background. I*r8a*^1^ mutant (*w*^−^*,Ir8a*^1^)^31^, *Ir8a* rescue (*w*^−^*, Ir8a*^*1*^*, p{Ir8a*^*+*^})^31^, and *Orco*^*1*^ rescue (*w*^−^*, Orco*^*1*^*, pBac{Orco*^*+*^**})^33^ were gifts from R. Benton. *Orco*^*1*^ mutant (*w*^−^*; + ; Orco*^*1*^)^30^, *Ir75a-Gal4* (*w*^−^*; + ; Ir75a-Gal4*)^33^ and *UAS-kir2.*1 (*w*^−^*; + ; UAS-kir2.1:eGFP*)^32^ flies were obtained from the Bloomington stock center.

### Food media

Flies were reared on Fly food medium containing agar (10 g/L), glucose (167 mM), sucrose (44 mM), yeast (35 g/L), cornmeal (15 g/L), wheat germ (10 g/L), soya flour (10 g/L), molasses (30 g/L), propionic acid and Tegosept. Flies were reared and assayed in a 12:12 hr light/dark cycle (LD 12:12) at 25 °C. Virgin adults were collected using CO_2_ anaesthesia and aged in same-sex groups of 20 in food vials for 5–8 days. To test individual food components, compounds at the same concentration as in fly food were boiled in water and agar (10 g/L) and supplemented with antibiotics propionic acid and Tegosept. 3 ml of food was poured in a 35 × 10 mm petri dish. Aspartame (Fischer Scientific), Sucralose and Na Saccharin (Sigma Aldrich), Arabinose and Sorbitol (Sigma Aldrich) were dissolved in 0.37% agar in water. Yeast amino acids (24, peptone or tryptone (BD Bioscience) were used at a concentration of 35 g/L. For the acetic acid treatment, 30 μl of acetic Acid was poured in a 35 × 10 mm petri dish and supplemented with 3 ml of food medium to reach a 1% acetic acid concentration.

### Mating assay

One virgin male and female (age 5–8 days old) were placed in a 35 × 10 mm petri dish layered with food media. For group mating assays, 6 virgin females followed by 6 virgin males were placed into a larger (55 × 13 mm) petri dish prepared as described above to keep flies at similar densities between the pair and group assay. All flies were aged on fly food and had *ad libitum* access to food prior to the experiments. All experiments began between Zeitgeber time (ZT) 7 and 8 and were housed in a temperature controlled chamber set at 25 °C in a 12:12 light:dark cycle. Red light was utilized to visualize the chambers during the dark phase. Pictures of the dishes were taken at 2-min intervals for 24 hr to score the number of copulations as described in^8^,^9^,^47^. A successful copulation was scored when a pair was seen *in copulo* in more than five successive frames (*D. melanogaster* mates for an average of 16 minutes).

### Yeast odour assay

A single colony of Baker’s Yeast (Redstar, Dutscher Scientific, UK LTD) was inoculated in a 1 L Erlenmeyer containing YPD medium 24 hours before the beginning of the experiment and cultured at 25 °C with constant stirring. Pressured air was used to bubble air through the yeast-culture. Before entering the yeast culture, the air was passed through an activated charcoal filter, located in a different room to avoid recycling experimental air, and a 0.15 μm filter to avoid transfer of microorganisms in the yeast culture. After the air exited the yeast culture it was passed through a fiberglass mesh filter to prevent any airborne yeast from entering the mating dishes. The air was split via a manifold and diverted into 40 mating dishes using Tygon tubing. Control air originated from the same pressured air system and bubbled through sterile YPD medium, and vented in the mating dishes as for the yeast air. The experiments were conducted in a bespoke stainless steel enclosure [63(D) × 71(H) × 120(L) cm]. The enclosure was cleaned with ethanol between experiments to avoid carry-over smells. The chamber was lit internally by white LED lights during the light phase of the day and red (>620 nm) LED lights during the dark phase. Flies were monitored in a 12:12 LD condition. The enclosure was equipped with air fans connected to an exhaust pipe extracting the yeast and control air outside the room.

### Progeny count

Eggs laid in the food during the 24 hr mating assay were transferred to a vial containing fly food and allowed to develop in this standard medium. The vials were maintained at 25 °C in a 12:12 light:dark cycle until adults eclosed and were counted.

### Statistical analysis

The effects of food and social context on reproductive performance were tested with mixed effects models using the lme4 package in R^48^. Food condition, air condition and genotype were included as fixed effects and tested for interactions. Because experiments were replicated over a number of days, we included date as random effect to account for day-to-day variability. We performed model selection by backwards elimination of non-significant fixed effects using log-likelihood ratio tests and the associated Akaike information criterion differences. In case of non-significant interactions, post-hoc analyses were performed to quantify the strength of the fixed effects per group. Residuals were visually inspected for normality and homogeneity of variances were evaluated with the Levene’s test. In case the residuals deviated from the normality assumption we used cumulative logit link models from the package Ordinal, which class of models can be regarded as an extended logistic regression, especially designed to analyze ordinal factor variables. In a few specific cases, we used non-parametric Kruskal-Wallis tests. Kruskal-Wallis, linear regressions and Spearman correlations were determined using GraphPad prism (GraphPad software Inc., Version 5.0 for Mac). To determine the effect size r, we determined the partial correlation coefficient of the effect of glucose on the number of copulations following equation 11 in^49^. We used the Z scores of the cumulative link models to determine r as follows: r = Z/sqrt(Z^2^ + df). Significant predictors are summarized in the figures or text and reported in detail in Table S1.

## Additional Information

**How to cite this article**: Gorter, J. A. *et al*. The nutritional and hedonic value of food modulate sexual receptivity in *Drosophila melanogaster* females. *Sci. Rep.* **6**, 19441; doi: 10.1038/srep19441 (2016).

## Supplementary Material

Supplementary Information
