# Supplementary material for: The nutritional and hedonic value of food modulate sexual receptivity in Drosophila melanogaster females
Source: Sci Rep. 2016 Jan 18;6:19441. doi: 10.1038/srep19441 (PMC4726014; doi:10.1038/srep19441)
Supplement: Supplementary Information [file srep19441-s1.pdf]

The nutritional and hedonic value of food modulate sexual receptivity in *Drosophila melanogaster* females

Jenke A. Gorter[1], Samyukta Jagadeesh[1,2], Christoph Gahr[1], Jelle J. Boonekamp[1], Joel D. Levine[2], and Jean-Christophe Billeter[1*].

[1] Groningen Institute for Evolutionary Life Sciences, PO Box 11103, University of Groningen, Groningen, 9700 CC, The Netherlands.

[2]Department of Biology, University of Toronto at Mississauga, 3359 Mississauga Road, Mississauga, ON, L5L 1C6, Canada.

*to whom correspondence should be addressed: email: j.c.billeter@rug.nl. Telephone: +31 50 363 7851.

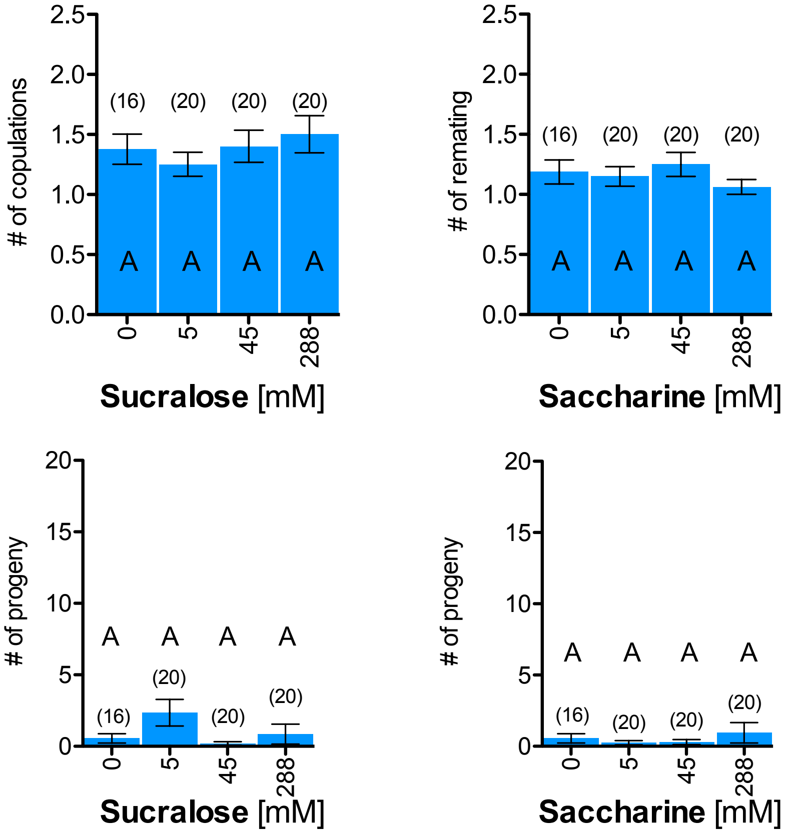

Figure S1. Mean number of copulations and progeny of one *Canton-S* female housed with a single male. Flies were provided the sweeteners Sucralose or Saccharine. concentration indicated in mM below the Y-axes). These sweeteners did not increase mating rate or progeny production. Bar graphs labeled with same letters are not significantly different from each other as determine by an ANOVA followed by Tukey's post-hoc test. Number of replicates is indicated above the bar graphs. Data represent Mean +/- S.E.M.

Food conditions influence receptivity and production of offspring (Fig 1)

Figure panel	Model factors	AIC	Explanatory variable	Estimate	Std. Error (+/-)	z value	p value**
	Model choice		Detailed model values				
A	Intercept	729.68					
	Food	582.92	Food	5.1875	0.4536	11.435	<0.001
	Food+Genotype	534.47	Genotype	-2.8988	0.3572	-8.115	<0.001
	**Food+Genotype+Group size**	**454.28**	Group size	-2.7338	0.3347	-8.168	<0.001

Smell of yeast affects female sexual receptivity via the ionotropic odourant receptor family (Fig 2)

Figure panel	Model factors	ChiSquare	p value*	AIC	Explanatory variable	Estimate	Std. Error (+/-)	t value
	Model choice				Detailed model values			
A	Intercept			284.56				
	Food	11.207	0.001	275.35	Intercept	4.9242	0.2872	17.145
	**Food+Air**	**3.5149**	**0.061**	**273.84**	Food	-0.1356	0.3824	-3.546
	Food+Air, Food*Air	1.9027	0.168	273.93	Air	0.6766	0.3641	1.858
Post-hoc	No factor			179.74	Intercept (food-yeast)	4.7500	0.3019	15.735
	Air	5.6297	0.018	176.11	Air (food-yeast)	1.0326	0.4315	2.393
	Intercept			99.589	Intercept (food+yeast)	3.9167	0.4734	8.274
	Air	1.00E-04	0.992	101.589	Air (food+yeast)	0.0064	0.6564	0.010
B	Intercept			444.25				
	Genotype	20.578	<0.001	425.67	Intercept	3.8466	0.3145	12.230
	**Genotype+Air**	**21.516**	**<0.001**	**406.15**	Genotype	-1.3533	0.2648	-5.111
	Genotype+Air, Genotype*Air	0.4744	0.491	407.68	Air	1.2794	0.2646	4.835
Post-hoc	Intercept			216.56	Intercept (*Orco1*)	3.9137	0.3527	11.100
	Air	7.5265	0.006	211.03	Air (*Orco1*)	1.1011	0.3905	2.820
Post-hoc	Intercept			214.19	Intercept (*Orco1,p{Orco}*)	2.3103	0.2588	8.928
	Air	14.134	<0.001	202.06	Air (*Orco1,p{Orco}*)	1.4674	0.3727	3.937
C	Intercept			419.84	Intercept	3.7548	0.3886	9.662
	Genotype	1.1164	0.291	420.72	Genotype	-0.3077	0.4711	-0.653
	Genotype+Air	5.2506	0.022	4.17.47	Air	0.1044	0.4759	0.219
	**Genotype+Air, Genotype*Air**	**3.9283**	**0.047**	**415.54**	Air*Genotype	1.3131	0.6665	1.970
Post-hoc	Intercept			187.34	Intercept (*Ir8a-*)	3.6862	0.4360	8.454
	Air	0.0672	0.796	189.27	Air (*Ir8a-*)	0.0996	0.3946	0.252
Post-hoc	Intercept			230.7	Intercept (*Ir8a-,p{Ir8a}*)	3.5000	0.3769	9.287
	Air	7.083	0.008	225.61	Air (*Ir8a-,p{Ir8a}*)	1.4259	0.5280	2.700

Acetic acid and proteins produced by yeast interact to modulate female reproductive behaviours (Fig 3)

Figure panel	Model factors	ChiSquare	p value*	AIC	Explanatory variable	Estimate	Std. Error (+/-)	t value	p value**
	Model choice				Detailed model values				
A	Intercept			247.52	Intercept	1.7484	0.3123	5.599	
	Food	1.7595	0.185	247.76	Food (Yaa vs No Yaa)	1.5000	0.3412	4.396	<0.001
	Food+Air	2.3934	0.302	249.37	Air (control vs AcAc)	0.2755	0.3041	0.906	0.368
	**Food+Air+Food*Air**	**16.585**	**<0.001**	**236.78**	Air (EA vs AcAc)	0.3749	0.4137	0.906	0.371
					Food (Yaa vs No Yaa)*Air (control vs AcAc)	-1.5833	0.3967	-3.991	<0.001
					Food (Yaa vs No Yaa)*Air (EA vs AcAc)	-1.7000	0.4825	-3.523	0.001
B1	Intercept			602.02	Intercept	1.7503	0.1725	10.149	
	Food	22.67	<0.001	585.35	Air	0.1901	0.1778	1.070	0.286
	Food+Air	19.639	<0.001	567.71	Food (peptone vs no food)	0.2379	0.2252	1.056	0.293
	**Food+Air+Food*Air**	**13.666**	**0.003**	**560.04**	Food (tryptone vs no food)	0.5661	0.3030	1.852	0.066
					Food (Yaa vs no food)	0.0667	0.2065	0.323	0.747
					Air*Food (peptone vs no food)	0.6307	0.2909	2.168	0.032
					Air*Food (tryptone vs no food)	0.7330	0.3858	1.900	0.060
					Air*Food (Yaa vs no food)	1.2017	0.3652	3.290	<0.001
B2	Interept			479.14	Intercept	1.6958	0.2736	6.197	
	Food	25.22	<0.001	459.92	Air	0.4607	0.3281	1.404	0.162
	Food+Air	16.279	<0.001	445.64	Food (peptone vs no food)	0.9015	0.3646	2.473	0.015
	**Food+Air+Food*Air**	**11.245**	**0.010**	**440.4**	Food (tryptone vs no food)	0.6714	0.5397	1.244	0.216
					Food (Yaa vs no food)	0.8881	0.3567	2.490	0.014
					Air*Food (peptone vs no food)	1.0806	0.4575	2.362	0.019
					Air*Food (tryptone vs no food)	0.6408	0.6409	1.000	0.319
					Air*Food (Yaa vs no food)	-0.6376	0.5577	-1.143	0.255
C1	Intercept			-1.7888	Intercept (*UAS-kir*)	1.0690	0.0477	22.440	
	Air	7.5758	0.006	-7.3646	AcAc (*UAS-kir*)	0.1875	0.0665	2.820	
	Intercept			28.227	Intercept (*Ir75a-Gal4*)	1.3944	0.0753	18.512	
	Air	13.331	<0.001	16.896	AcAc (*Ir75a-Gal4*)	0.3012	0.0769	3.917	
	Intercept			-11.0966	Intercept (*Ir75a-Gal4>UAS-kir*)	1.0666	0.0432	24.700	
	Air	0.6011	0.438	-9.9677	AcAc (*Ir75a-Gal4>UAS-kir*)	0.0464	0.0611	0.760	
C2	Intercept			306.83	Intercept (*UAS-kir*)	18.7810	10.0260	1.873	
	Air	2.5003	0.114	306.33	AcAc (*UAS-kir*)	6.5570	4.1030	1.598	
	Intercept			403.85	Intercept (*Ir75a-Gal4*)	14.7470	5.8930	2.502	
	Air	21.614	<0.001	384.24	AcAc (*Ir75a-Gal4*)	19.3440	3.6910	5.241	
	Intercept			373.22	Intercept (*Ir75a-Gal4>UAS-kir*)	21.5530	4.0400	5.335	
	Air	8.3321	0.004	366.89	AcAc (*Ir75a-Gal4>UAS-kir*)	13.3220	4.3040	3.095	

Taste and calorific value of sugars interact to modulate female reproductive behaviours (Fig 4)

Figure panel	Model factors	AIC	Explanatory variable	Estimate	Std. Error (+/-)	z value	p value**
	Model choice		Detailed model values				
A1	Intercept	108.21					
	Aspartame concentration	110.21	Aspartame concentrations	0.0001	0.0021	0.033	0.973
A2	Intercept	150.75					
	Aspartame concentration	151.46	Aspartame concentrations	-0.0028	0.0026	-1.078	0.281
B1	Intercept	285.3					
	Genotype	255.91					
	**Genotype+Glucose concentration**	**233.37**	Glucose concentration	0.0073	0.0016	4.667	<0.001
	Genotype+Glucose+Genotype*Glucose	235.37	Genotype	-2.7038	0.4934	-5.480	<0.001
Post-hoc	Intercept	196.48					
	Glucose concentration	180.64	Glucose concentration (*Canton-S*)	0.0071	0.0018	4.020	<0.001
Post-hoc	Intercept	60.26					
	Glucose concentration	55.94	Glucose concentration (*Gr64a-*)	0.0075	0.0033	2.271	0.0232
B2	Intercept	683.18					

	Genotype	678.49					
	**Genotype+Glucose concentration**	**646.47**	Glucose concentration	0.0072	0.0013	5.558	<0.001
	Genotype+Glucose+Genotype*Glucose	646.53	Genotype	-1.1342	0.3439	-3.297	0.001
Post-hoc	Intercept	414.66					
	Glucose concentration	383.56	Glucose concentration (*Canton-S*)	0.0097	0.0018	5.327	<0.001
Post-hoc	Intercept	248.2					
	Glucose concentration	244.66	Glucose concentration (*Gr64a-*)	0.0043	0.0019	2.309	0.021
Figure panel	Model factors	AIC	Explanatory variable	Estimate	Std. Error (+/-)	t value	p value**
	**Model choice**		**Detailed model values**				
C1	Intercept	150.46	Intercept	1.6429	0.2134	7.700	0.000
	Food	141.93	Food(Arabinose vs agar)	0.2238	0.2967	0.754	0.454
			Food(Sorbitol vs agar)	-0.2429	0.2967	-0.819	0.417
			Food(Arabinose+Sorbitol vs agar)	0.8956	0.3075	2.913	0.005
C2	Intercept	336.38	Intercept	4.0000	1.2048	3.320	0.002
	Food	339.28	Food(Arabinose vs agar)	1.0667	1.6752	0.637	0.527
			Food(Sorbitol vs agar)	-0.6667	1.6752	-0.398	0.692
			Food(Arabinose+Sorbitol vs agar)	2.3077	1.7363	1.329	0.190

Supplementary table S1 - Summary statistical analysis

All models in this table were performed using R version 3.2.2. All data that complied with the rules of normality and homogeneity (either before or after square-root transformation) were tested with mixed effects linear models and the output is shown here with t- and p-values. When either normality or homogeneity could not be satisfied, a cumulative link model was used and the output is shown here with z- and p-values. When applicable a random effect for date was added to the models.
*Log likelihood ratio test **Test statistics (contingency table convert)
